# Challenges in Managing Patients with Hereditary Cancer at Gynecological Services

**DOI:** 10.1155/2019/4365754

**Published:** 2019-05-27

**Authors:** Mako Ueda, Hiroshi Tsubamoto, Mina Kashima-Morii, Yoshitaka Torii, Mariko Kamihigashi, Yu Wakimoto, Nami Nakagomi, Tomoko Hashimoto-Tamaoki, Hideaki Sawai, Hiroaki Shibahara

**Affiliations:** ^1^Department of Obstetrics and Gynecology, Hyogo College of Medicine, Nishinomiya, Japan; ^2^Department of Clinical Genetics, Hyogo College of Medicine, Nishinomiya, Japan; ^3^Department of Surgical Pathology, Hyogo College of Medicine, Nishinomiya, Japan

## Abstract

**Aim:**

To reveal current problems and challenges faced by our gynecologic services department in managing patients with hereditary cancers.

**Methods:**

We collected clinical data of patients with hereditary cancers, identified via genetic testing (or clinically diagnosed in cases of Cowden syndrome or Peutz–Jeghers syndrome), and treated in our gynecological department from 2012 to 2018.

**Results:**

Fifteen patients had hereditary breast and ovarian cancer (HBOC), 6 had Lynch syndrome, 2 had Cowden syndrome, and 2 had Peutz–Jeghers syndrome. Five patients diagnosed with HBOC were younger than 40 years at diagnosis. Risk-reducing salpingo-oophorectomy (RRSO) was performed on 1 patient with a *BRCA1* mutation at age 38 years. Seven patients overall underwent RRSO, and none had malignancies on pathological examinations. Peritoneal washing cytology (PWC) was suspicious for malignancy in one patient; however, subsequent PWC at 6 months after RRSO was negative. A patient with endometrial cancer and Lynch syndrome and a patient with atypical endometrial hyperplasia (AEH) and Cowden syndrome strongly desired fertility preservation. They achieved remission after medroxyprogesterone acetate treatment and multiple dilations and curettages, respectively. One patient with Lynch syndrome developed AEH after 11 years of surveillance. Laparotomy revealed adjacent low-grade and high-grade serous ovarian cancer with positive ascites cytology. She had no recurrence during 7-year follow-up after laparotomy.

**Conclusion:**

Managing patients with hereditary cancer, positive or false-positive ascites cytology discovered during RRSO, and desired preservation of fertility is highly challenging.

## 1. Introduction

Hereditary cancer comprises a group of diseases caused by gene mutations that are associated with risks of synchronous, metachronous, and multiorgan cancer. Affected patients need to be identified, managed by a multidisciplinary team, and placed under periodic surveillance for cancer treatment and prevention. The Familial Tumor Center was established at our hospital to conduct family surveys, registrations, and gene analyses from 2001 to 2009. A multidepartment coordination system was established to accommodate the needs of patients and families after the assignment of certified genetic counselors began in 2011 [1]. Familial Tumor Data Sharing in our hospital, risk-reducing salpingo-oophorectomy (RRSO) for patients with *BRCA* gene mutations, and regional coordination of hereditary breast and ovarian cancer (HBOC) management were instituted upon approval of the ethical review board (no. 187, no. 256, and no. 274, respectively). More patients with hereditary cancers identified via genetic testing are now being treated in the gynecological services department after the establishment of the coordination system. As such, we have taken on some difficult cases in daily practice.

In Japan, the Basic Plan to Promote Cancer Control Programs was revised in March 2018, which promoted genomic cancer medicine programs. Increasing numbers of patients have requested sequencing tumor genomes including germline mutations. The use of poly ADR-ribose polymerase (PARP) inhibitor, olaparib, approved for recurrent breast cancer in July 2018, requires a concurrent companion diagnostic test for pathogenic *BRCA* mutations (variants). The frequency of treating patients with hereditary cancer at gynecological services is thus expected to increase rapidly. We report the current practice at our clinic and the challenges we have faced in managing these patients.

## 2. Methods

We collected the clinical data of patients with hereditary cancer who were identified via genetic testing or were clinically diagnosed with Cowden syndrome or Peutz–Jeghers syndrome and who were treated in the gynecological services department from 2012 to 2018. The patients were also registered in the Familial Tumor Data Sharing system. We also listed the more difficult cases individually. Informed consent was obtained from each of the patients for inclusion in this research (the ethical review board of no. 187).

## 3. Results

Fifteen patients (13 families) had HBOC, 6 had Lynch syndrome, 2 had Cowden syndrome, and 2 had Peutz–Jeghers syndrome. None of the patients had Li–Fraumeni syndrome (Table 1). Of the 15 patients with HBOC, 8 were tested at our clinic and 7 were tested at other clinics. Six patients were *BRCA1* positive, and 9 patients were *BRCA2* positive. Seven of the 15 patients with HBOC (2 *BRCA1* positive and 5 *BRCA2* positive) underwent RRSO. One of the patients is scheduled for bilateral salpingo-oophorectomy (BSO) for an ovarian cyst. The mean age of the patients at the time of RRSO was 53 years. No occult cancers or serous tubal intraepithelial carcinomas (STIC) were found during RRSO. One patient had positive ascitic cytology suspicious for malignancy. Seven patients who have not undergone RRSO are currently under surveillance, and their mean age was 39 years.

Lynch syndrome was diagnosed based on genetic testing in 6 patients. Three of them were tested at our clinic. Three patients had mutL homolog 1 (*MLH1*), 1 had mutS homolog 2 (*MSH2*), 1 had mutS homolog 6 (*MSH6*), and 1 patient's result was not disclosed. The mean age at the time of diagnosis was 38 years. One patient (no. 20) was diagnosed with Lynch syndrome after resection of sigmoid colon cancer in her 30s. Atypical endometrial hyperplasia (AEH) was found after 11 years of surveillance. Two tumors adjacent to the fallopian tube and adherent to the ovarian surface were identified during laparotomy, necessitating hysterectomy and bilateral salpingo-oophorectomy (BSO). These were pathologically diagnosed as low-grade and high-grade serous cancers with no apparent stromal invasion [2]. The patient has been followed without treatment, despite having positive ascitic cytologies, and has had no evidence of recurrence at 7 years after hysterectomy and BSO. Two patients were diagnosed with Cowden syndrome, one was diagnosed based on genetic testing, and another was diagnosed clinically. Peutz–Jeghers syndrome was diagnosed in 1 patient in our clinic based on the presence of gastric hamartomatous polyposis and mucocutaneous pigmented spots.

### 3.1. Difficult Cases

#### 3.1.1. Positive Ascites Cytology at the Time of RRSO (Patient No. 6)

**Figure 1:**
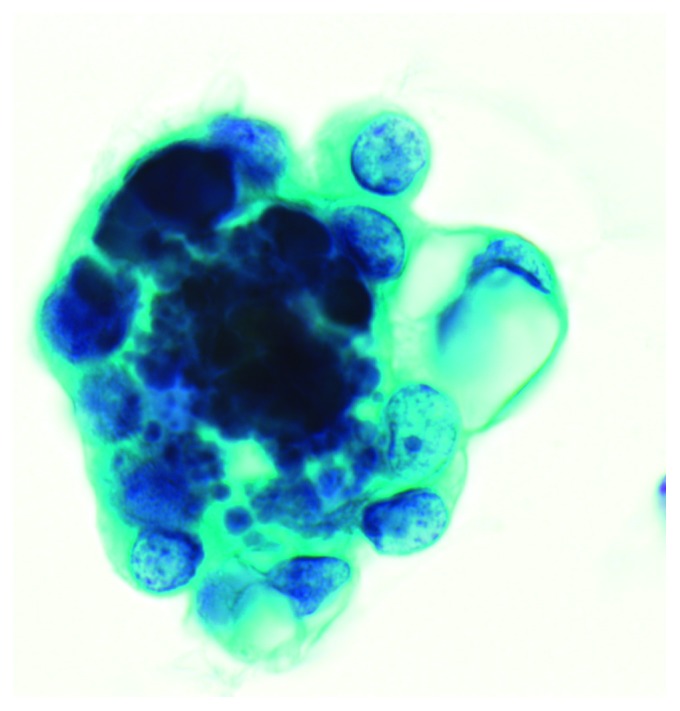
Ascites cytology after RRSO. An atypical cell cluster is seen. Atypical cells show unevenly distributed nuclei, overlapping nuclei, anisonucleosis, and prominent nucleoli. RRSO: risk-reducing salpingo-oophorectomy.

This patient was referred to our clinic by two of her physicians, an oncologist and a gynecologic oncologist, after her mother (Patient no. 16) underwent RRSO. There were no abnormalities on her preoperative examination. There were no findings of malignancy or endometriosis upon surveillance of the pelvis during laparoscopy. A detailed pathological examination of the excised ovary and fallopian tube was negative for malignancy; however, the peritoneal washing cytology (PWC) indicated suspected adenocarcinoma (Figure 1). Further examination of a paraffin-embedded sample was also negative for malignancy. After an assessment by an outside cytopathologist and consensus with the referring oncologist and gynecologic oncologist, a positron emission tomography (PET) scan was performed, followed by a second-look laparoscopy 6 months later. The PET scan found no abnormality. No macroscopic abnormalities were observed during second-look laparoscopy. Biopsies were taken from 3 different parts of the peritoneum in the pouch of Douglas, and peritoneal washing ascites was collected. No abnormalities were identified in any of the samples.

#### 3.1.2. Fertility Preservation in a Patient with Endometrial Cancer Associated with Lynch Syndrome (Patient No. 19)

An unmarried nulligravida in her 30s with endometrial cancer was referred to our clinic in consideration of fertility preservation because of an extensive family history suggestive of Lynch syndrome. The patient did not wish to undergo genetic testing. After being thoroughly informed of the possibility of Lynch syndrome based on relevant study reports, she consented to medroxyprogesterone acetate (MPA) therapy at 600 mg/day for 6 months. She ultimately achieved remission. A right triple negative breast cancer (TNBC) developed 2 years later. At that time, she requested genetic testing, which was required for developing a treatment protocol to manage the breast cancer and promote fertility preservation. Lynch syndrome was diagnosed based on the pathogenic *MLH1* mutation. Both ovaries and oocytes were cryopreserved prior to neoadjuvant chemotherapy. She has had no endometrial cancer recurrence at 3 years after MPA therapy.

#### 3.1.3. Fertility Preservation in a Patient with AEH Associated with Cowden Syndrome (Patient No. 22)

An unmarried nulligravida in her 30s had a left breast cancer in her 20s. Breast-conserving surgery was performed followed by tamoxifen therapy. The patient underwent genetic testing because her father had been diagnosed with Cowden syndrome. A pathogenic mutation of the phosphatase and tensin homolog (*PTEN*) gene was identified. The patient had thyroid cancer in her 30s and had a thyroidectomy. The right breast cancer developed around the same time. She underwent breast-conserving surgery and was administered gonadotropin-releasing hormone (GnRH) agonist and tamoxifen therapy. She consulted our gynecological services department and requested to be included in cross-department surveillance at our hospital. Atypical endometrial hyperplasia was diagnosed 2 years later, and the tamoxifen was discontinued. The patient underwent dilation and curettage (D&C) every 6 months thereafter. No recurrence of AEH has been reported in the past 5 years.

#### 3.1.4. Anxieties after Disclosure of a Pathogenic Mutation (Patient No. 5)

The patient was seen in the Department of Breast Surgery with a lump she identified during a self-breast exam when she was in her 30s. Biopsy at that time revealed TNBC. The patient expressed her concerns about the possibility of hereditary cancer during a preoperative chemotherapy session. She was concerned because her mother had also been diagnosed with breast cancer. She was referred to the Department of Genetic Counseling. Total mastectomy of the right breast and sentinel lymph node dissection were performed. The patient had several postoperative counseling sessions and requested a genetic test 9 months after the first counseling session. After a pathogenic *BRCA1* mutation was found, the patient developed new anxieties as a result of the unexpected diagnosis and her history of the other type of cancer. Following multiple counseling sessions and surveillance by our gynecological services department, she underwent RRSO at 7 months after disclosure of the genetic test result. No pathological abnormalities were identified.

## 4. Discussion

The number of patients with genetically diagnosed hereditary cancer who were treated at our gynecological services department from 2012 to 2018 seemed small. In Ontario, where free *BRCA* screening services are provided by the municipal government/healthcare institutions, only 6.6% of patients with serous ovarian cancers were screened between 2001 and 2011 [3]. In our gynecological services department, outpatients with gynecological cancers were interviewed by genetic counselors from 2012 to 2016. Among patients with high risk of inherited cancer, 8.9% underwent genetic testing. Pathogenic variants were observed in 40% of patients who underwent genetic testing [1]. The reasons for not wanting to be tested included the following: testing would not cure the current disease (e.g., the test would not affect the current treatment); the patient wanted to concentrate on the current cancer treatment; the patient did not want to worry her family; and financial burden from treatments not covered by health insurance. Few patients named the lack of relevant laws such as the Genetic Information Nondiscrimination Act and the Health Insurance Portability and Accountability Act in the United States of America (U.S.A.) as a reason. The rationale for actively recommending genetic counseling was lacking because the incidence of hereditary gynecological cancer in Japan was previously unknown. However, the hereditary cancer incidence in Japan is now known to be comparable to that in the U.S.A. or Europe. This is based on recent studies which reported that the incidence of HBOC among ovarian cancer patients was 11.8%, the incidence of Lynch syndrome among ovarian cancer patients was 2.6%, and the incidence of Lynch syndrome among endometrial cancer patients was 4.4% [4, 5].

Current problems and challenges in managing hereditary cancer patients in the gynecological services department are discussed below.

### 4.1. Anxieties after Disclosure of Pathogenic Mutation

Genetic counseling for cancer generally eliminates the patient's anxieties [6]. We encouraged self-determination in many of our patients through multiple counseling sessions. As a result, most patients were able to take the subsequent treatment and surveillance in stride. However, it required a long time for Patient no. 5 to accept an unexpected genetic test result, which was administered after multiple counseling sessions held over a 9-month period. Although the overly anxious personality of this patient may have been a significant factor, her anxieties were multifaceted and vague, since elimination of the anxieties was the reason for the first consultation. The final course of treatment was undetermined at the time of the first consultation, at which point she was undergoing preoperative chemotherapy for breast cancer. The potential for good news was the primary reason for this patient to undergo counseling; receiving accurate information and deepening her knowledge did not help eliminate her anxieties. The lack of a characteristic family history also increased her expectations of receiving good news. The patient was extremely shocked to learn that the genetic testing showed a pathogenic *BRCA1* mutation, which she had not expected. It was difficult for her to accept the test result. We recommended RRSO during outpatient surveillance, and the procedure was performed 7 months after the disclosure of the genetic test result.

Her case shows us that determining the patient's reason for consultation and the current disease and treatment are both fundamental and extremely important in genetic counseling. Although presenting the results of assessments made using risk prediction tools based on patients' family history is general practice, genetic counseling should be neutral irrespective of the assessment results. Thorough counseling on the possibility of unexpected results is important.

### 4.2. Management of HBOC Patients under 40

The ideal timing for RRSO to prevent ovarian cancer in patients with HBOC who are under the age of 40 is controversial. Until 2017, the National Comprehensive Cancer Network (NCCN) guidelines stated that the ideal age at which to undergo RRSO was from 35 to 40 years of age, after the patient has had children, regardless of *BRCA* status; however, the guidelines were revised and now RRSO can be delayed until the patient reaches 40 to 45 years old, since the age of ovarian cancer onset in *BRCA2*-positive patients is 8 to 10 years older than the mean age of onset in *BRCA1*-positive patients. The attending physicians should stay current with this type of information.

Hormone replacement after RRSO is acceptable based on a report on hormone replacement, in which the authors stated that hormone replacement therapy does not reverse the effects of RRSO as it reduces breast cancer risks. Short-term hormone therapy is acceptable as long as it is discontinued before the usual age of menopause. However, hormone therapy should be given carefully since no data are available from randomized controlled trials [7].

Prophylactic salpingectomy with delayed oophorectomy (PSDO) has also been considered as a possible treatment because more than 80% of ovarian cancers in *BRCA* mutation carriers are of the serous type, and more than 90% of serous ovarian cancers may be high-grade serous carcinoma (HGSC) originating from the tubal epithelium [8, 9]. The NCCN guidelines do not consider PSDO to be a standard treatment because its prophylactic effects are unproven. Problems with PSDO include the residual risk of ovarian cancer and the smaller effect of reducing breast cancer risk in premenopausal women compared with RRSO. A clinical study to evaluate the effects of PSDO is ongoing.

Five of our patients with HBOC included were under the age of 40 at the time of diagnosis (Table 2). The patients received thorough counseling on prophylactic surgery based on the NCCN guidelines and the previously mentioned studies. Only one of the 5 patients (Patient no. 5) was a pathogenic *BRCA1* mutation carrier. She underwent RRSO at the age of 38. The other 4 patients were carriers of pathogenic *BRCA2* mutations, with no third-degree relative having been diagnosed with ovarian cancer. These patients are under periodic surveillance without plans for prophylactic surgery.

### 4.3. Post-RRSO False-Positive Ascites Cytology

Intraoperative macroscopic findings during RRSO or histopathological examination of resected samples may unexpectedly suggest malignant or precancerous lesions. Detailed histopathology according to the sectioning and extensively examining the fimbriated end (SEE-FIM) protocol and postoperative evaluation by a gynecologic oncologist and a pathologist have been considered necessary. However, outcomes among patients with positive PWC (malignant or suspicious for malignancy) without pathologically identified malignancy or precancerous lesions have not been reported frequently. In the literature, 11 patients have had positive PWC with no macroscopic malignancies found during RRSO (Table 2) [10–14]. The frequency of cases with no macroscopic findings consistent with malignancy but with positive PWC is about 1%, which is not low. One patient with a tumor classified as suspicious for malignancy had endometriosis and endosalpingiosis and was followed after RRSO [11]. Microscopic malignancy or STIC was found in 5 patients. The other 5 patients had positive PWC without pathologically identified malignancies or precancerous lesions. Three of them received postoperative chemotherapy. Patient no. 6, who had positive PWC without macroscopic abnormality or evidence of pathological malignancy during RRSO, subsequently had negative PWC at 6 months and was followed closely.

### 4.4. Fertility Preserving Treatment in Patients with Lynch or Cowden Syndrome and Endometrial Cancer or Precancerous Lesion

Currently, fertility preservation is quite an important issue with respect to cancer treatment in young women. The American Society of Clinical Oncologists (ASCO) issued a recommendation for fertility preservation in cancer patients in 2006. The 2018 ASCO recommendation states, “People with cancer are interested in discussing fertility preservation. Health care providers caring for adult and pediatric patients with cancer should address the possibility of infertility as early as possible before treatment starts.” In Japan, the Japanese Society for Fertility Preservation issued the “Guide for pregnancy, delivery, and reproduction medicine in breast cancer patients” in 2014, and the Japan Society of Clinical Oncology issued the “Treatment guidelines for fertility preservation in pediatric, adolescent, and young cancer patients” in 2017. Regional oncofertility networks are being established nationwide. In 2016, we established the Hyogo oncofertility network with the Center for Reproductive Medicine as the core institution that would actively advocate fertility preservation in young cancer patients. Fertility preservation in hereditary cancer patients brings many challenges. Few reports are available, especially as it relates to fertility preservation in patients with endometrial cancer.

Only one case of attempted fertility preservation with high-dose progestin in a young patient with endometrial cancer associated with Lynch syndrome has been reported [15]. Despite the strong family history of Lynch syndrome, the patient had not been genetically tested. A genetic diagnosis was made after fertility preservation treatment. One patient (no. 19) requested fertility preservation after receiving thorough information; however, whether to use MPA in a patient with endometrial cancer and confirmed Lynch syndrome should be considered carefully. Some prospective clinical studies of fertility preservation treatments in young patients with endometrial cancer excluded patients with suspected Lynch syndrome based on their family history [16]. One patient (no. 19) was diagnosed with a breast cancer after MPA therapy. The patient wished to preserve her fertility prior to starting preoperative chemotherapy. Lynch syndrome was suspected based on her family history and the onset of endometrial and breast cancer. Oocytes and unilateral ovarian tissue were cryopreserved after genetic testing to ensure that the ovarian tissue cryopreservation would be practical (to deny HBOC). The other ovary was preserved based on a study reporting no difference in ovarian cancer risk between young patients with endometrial cancer alone and those with concurrent Lynch syndrome [17]. Although no clinical report on the association between short-term high-dose progesterone therapy and the onset of breast cancer is available, the possibility of breast cancer should be taken into consideration when prescribing MPA therapy to patients with Lynch syndrome.

Complications with endometrioid adenocarcinoma and AEH have been reported, each occurring in one gynecological patient with Cowden syndrome and a pathogenic *PTEN* mutation [18, 19]. We found no reports on fertility preservation in patients with confirmed precancerous lesions. The lifetime risk of endometrial cancer in patients with Cowden syndrome and a pathogenic *PTEN* mutation is 28% [20]. We informed 1 patient (no. 22) of the risks involved in prior oral tamoxifen treatment after breast cancer surgery and with being a carrier of a pathogenic *PTEN* mutation. We explained that MPA therapy was impractical and hysterectomy might be considered. The patient wished to preserve her fertility after being thoroughly informed and having undergone counseling. Oral tamoxifen was discontinued, and complete D&Cs have been performed every 3 months for endometrial tissue examination. The patient has been receiving periodic examinations to date. The current examination frequency is every 6 months since the histopathology in the second and subsequent examinations all showed simple endometrial hyperplasia.

### 4.5. Surveillance in Patients with Lynch Syndrome

Early detection of ovarian cancer is difficult in the surveillance of patients with HBOC because of the peritoneal dissemination of HGSCs in the early stages. Møller et al. reported a 5-year survival rate of 88% in 19 Lynch syndrome patients with ovarian cancer onset during the surveillance period [21]. Woolderink et al. suggested that one of the reasons for the early detection of stage I or II ovarian cancer in 12 patients could be that an occult cancer was found during a hysterectomy for endometrial cancer or AEH [22]. Another reason for the early detection may be that HGSC is not a frequently occurring epithelial ovarian cancer associated with Lynch syndrome (endometrioid, 45%; high-grade serous, 28%; and low-grade serous, 13%) unlike most sporadic ovarian cancers or ovarian cancer associated with HBOC. The significance of Lynch syndrome surveillance can be seen in one patient (no. 20) who developed AEH after a 10-year surveillance period. At that time, she underwent hysterectomy during which low-grade and high-grade serous occult cancers were identified. She remained disease-free at 7 years after the hysterectomy.

While hereditary cancer treatments have advanced greatly in recent years, we often have a difficult time treating patients because little evidence is available from large-scale studies. Many of the patients are adolescents or young adults who need support with school, employment, or getting married in addition to medical care; however, regional support systems are often not available. The experience of our gynecologic services department, which we described in this paper, could provide useful information for managing patients with hereditary cancer.

A study on the role of genetic counselors in fertility preservation among patients with hereditary cancers has been submitted separately.

## Figures and Tables

**Table 1:** Demographics of patients with hereditary cancer treated in our gynecological services department between 2012 and 2018.

	Case no.	Genetic diagnosis					Cancer	Family history	Gynecological management
		Age at diagnosis	Pathogenic mutation	Mutation point	Refseq ID	dbSNP rs no.			
HBOC	1	20s	*BRCA2*	c.(7435 + 1_7436-1)_(7805 + 1_7806-1)dup	NM_000059.3	NA	Breast		Surveillance
	2	30s	*BRCA2*	c.188T > A	NM_007294.3	rs80357086	Breast	Breast cancer, mother	Surveillance
	3	30s	*BRCA2*	c.5576_5579delTTAA (p.Ile1859Lysfs)	NM_000059.3	rs80359520	Breast		Surveillance
	4	30s	*BRCA2*	p.S1882X	NM_000059.3	NA	Breast		Surveillance
	5	30s	*BRCA1*	c.5154G > A (p.W1718X)	NM_007294.3	rs80357239	Breast	Breast cancer, mother	RRSO (7 months after genetic diagnosis)
	6	40s	*BRCA2*	c.2T > G (p.M1R)	NM_000059.3	rs80358547	None	Breast cancer, mother (BRCA2, case 14)	RRSO (6 months after genetic diagnosis)
	7	40s	*BRCA1*	c.3122C > G (p.Ser1041Ter)	NM_007294	rs397509035	Breast	Bilateral breast cancer, mother Breast cancer, aunt	Surveillance
	8	50s	*BRCA1*	Not available	—	—	Ovarian	Breast cancer, sister	Surveillance
	9	50s	*BRCA2*	c.9117G > A (p.P3039P)	NM_000059.3	rs28897756	Bilateral breast Gastric	Breast cancer, maternal side Ovarian cancer, many relatives	RRSO (7 months after genetic diagnosis)
	10	50s	*BRCA2*	c.5574_5577delAATT (p.Ile1859Lsyfs*∗*3)	NM_000059	rs80359520	Breast Sigmoid colon	Sigmoid colon cancer, father Gastric cancer, aunt Breast and colon cancer, paternal grandmother	Surveillance
	11	50s	*BRCA2*	c.5645C > A (p.Ser1882Ter)	NM_000059	rs80358785	Breast	Ovarian cancer, mother	RRSO (4 months after genetic diagnosis)
	12	50s	*BRCA2*	c.5645C > A (p.Ser1883Ter)	NM_000059	rs80358785	Breast	Breast and ovarian cancer, mother	RRSO (3 months after genetic diagnosis)
	13	60s	*BRCA2*	c.4085delA	NM_000059.3	rs431825315	Breast	Breast cancer, father Breast and ovarian cancer, sister	RRSO (8 months after genetic diagnosis)
	14	60s	*BRCA1*	c.2T > G (p.M1R)	NM_000059.3	rs80358547	Breast	Breast cancer, mother and sister Ovarian cancer, grandmother and aunt	RRSO (8 months after genetic diagnosis)
	15	60s	*BRCA1*	c.3122C > G (p.Ser1041Ter)	NM_007294	rs397509035	Breast	Breast cancer, sister Breast cancer, daughter (case 7)	BSO scheduled for ovarian cyst

Lynch syndrome	16	20s	*MSH2*	p.Q130X	NM_000251.2	rs1060501989	Colon	Unknown	Surveillance
	17	20s	*MSH6*	c.3663delGAinsT	NA	NA	None	Colon, endometrial, ovarian cancer, mother (MSH6)	Surveillance
	18	20s	*MLH1*	c.381_543del (p.Ala128Trpfs*∗*8)	NM_000249.3	NA	None	Colon cancer, father (MLH1)	Surveillance
	19	30s	*MLH1*	c.306+1G > A (p.K70_Q102del)	NM_0000249	rs267607734	Endometrial Breast	Breast, ovarian, colon cancer, mother	MPA
	20	40s	*MLH1*	g.IVS3+1G > A	NA	NA	Sigmoid colon AEH, ovarian	Colon cancer, father (MLH1) and grandmother Colon and endometrial cancer, sister	TAH＋BSO
	21	70s	Not disclosed				Breast Colon Endometrial		TAH

Cowden syndrome	22	20s	*PTEN*	c.1026+1G > A	NM_000314	rs786201041	Bilateral breast Thyroid AEH	Father (*PTEN*)	D&C
	23	70s	Clinical diagnosis				Breast Thyroid	Bladder cancer, father Gastric and endometrial cancer, sister	Surveillance

Peutz–Jeghers syndrome	24	Teen	Clinical diagnosis	In house			Gastric		Surveillance
	25	50s	Clinical diagnosis	Others			None		Surveillance

HBOC: hereditary breast and ovarian cancer; RRSO: risk-reducing salpingo-oophorectomy; TNBC: triple negative breast cancer; AEH: atypical endometrial hyperplasia; TAH: total abdominal hysterectomy; BSO: bilateral salpingo-oophorectomy; D&C: dilatation and curettage; MPA: medroxyprogesterone acetate; *PTEN*: phosphatase and tensin homolog; and *BRCA*: breast cancer susceptibility gene.

**Table 2:** “Malignant” or “suspicious for malignancy” on peritoneal washing cytology without macroscopic abnormality.

	Period of RRSO	SEE-FIM protocol	PWC in RRSO	Malignancy in PWC (macroscopic cancer)	Histopathologic examination	Further examination and follow-up therapy
Colgan [10]	NA	No	35	Malignant	Early stromal invasion of the ovarian cortex	Chemotherapy, 2nd look
				Malignant	STIC	Observation
				Malignant	NED	Extensive additional sectioning, reviewed by additional 2 cytopathologists, negative cytology at 2nd look, chemotherapy and 2nd look surgery

Manchanda [11]	2005–2009	No	308	Malignant	STIC	Hysterectomy, OMT + LND
				Malignant	STIC	Hysterectomy, OMT + LND
				Suspicious for malignancy	STIC	Hysterectomy, OMT + LND

Landon [12]	2000–2012	Mostly	117	Malignant	NED	Reviewed by additional 4 cytopathologists, multiple levels of tissue blocks examined, chemotherapy, 2nd look surgery
				Suspicious for malignancy	Ovarian endometriosis and endosalpingiosis	Observation

Powell [13]	1995–2009	NA	405	Malignant	NED	Hysterectomy, OMT
				Malignant	NED	Hysterectomy, OMT followed by 6 cycles of chemotherapy
				Malignant	NED	Hysterectomy, OMT + LND followed by 6 cycles of chemotherapy

Blok [14]	2000–2014	Since 2006	267	None		

Total			1132	11 (1.0%)		

SEE-FIM: sectioning and extensively examining the fimbriated; PWC: peritoneal washing cytology; and STIC: serous tubal intraepithelial carcinoma.

## Data Availability

The data used to support the findings of this study are available from the corresponding author upon request.
